# Three-dimensional volumetric muscle reconstruction of the *Australopithecus afarensis* pelvis and limb, with estimations of limb leverage

**DOI:** 10.1098/rsos.230356

**Published:** 2023-06-14

**Authors:** Ashleigh L. A. Wiseman

**Affiliations:** McDonald Institute for Archaeological Research, University of Cambridge, Cambridge, UK

**Keywords:** hominin, biomechanics, moment arm, muscle modelling, musculoskeletal

## Abstract

To understand how an extinct species may have moved, we first need to reconstruct the missing soft tissues of the skeleton, which rarely preserve, with an understanding of segmental volume and muscular composition within the body. The *Australopithecus afarensis* specimen AL 288-1 is one of the most complete hominin skeletons. Despite 40+ years of research, the frequency and efficiency of bipedal movement in this specimen is still debated. Here, 36 muscles of the pelvis and lower limb were reconstructed using three-dimensional polygonal modelling, guided by imaging scan data and muscle scarring. Reconstructed muscle masses and configurations guided musculoskeletal modelling of the lower limb in comparison with a modern human. Results show that the moment arms of both species were comparable, hinting towards similar limb functionality. Moving forward, the polygonal muscle modelling approach has demonstrated promise for reconstructing the soft tissues of hominins and providing information on muscle configuration and space filling. This method demonstrates that volumetric reconstructions are required to know where space must be occupied by muscles and thus where lines of action might not be feasible due to interference with another muscle. This approach is effective for reconstructing muscle volumes in extinct hominins for which musculature is unknown.

## 1. Introduction

Soft tissues rarely preserve in the fossil record, rather we are mostly left with just the skeletal material. Yet, muscles animate the body. They allow an animal to move, walk and run. To understand how an extinct species may have moved, we first need to reconstruct the missing soft tissues of the skeleton with an understanding of volume and the composition within the body. While these challenges in reconstructing hominin musculature have been addressed to an extent in the past [1–8], prior studies have typically relied upon estimated attachment sites consisting of a singular landmark and a simple action line representing leverage of the muscle (e.g. [4]; although see [2]). However, with the development of advanced computational methods, more sophisticated muscle modelling techniques have advanced musculature estimates of functionality (e.g. [5]).

Although more recent methods are still founded in modelling lines of action which represent a muscle's volume and path [9,10], these action lines are more sophisticated than earlier studies (e.g. [1]) by the utilization of *via points* and *wrapping surfaces* [11,12]. These computationally advanced models produce well-validated estimates of limb functionality and leverage of a given extant species, which have proven extremely informative [13–16], but such modelling relies upon *known* muscle configurations within a limb, which is obviously problematic for musculoskeletal modelling of extinct species (e.g. [17,18]). Previous studies that have used sensitivity analyses during the muscle modelling process have demonstrated that changes in attachment site and path throughout a body can influence moment-generating capacity and/or moment arm values [12,13,16,19–21]. If the aim of the study is to address movement in a known specimen/individual, it is preferential for biomechanical models to be subject-specific and founded in known muscular compositions obtained via imaging methods [11,12,22], albeit this is a momentous and challenging task for modelling the musculature of an extinct species in which this is *unknown*.

To be able to elucidate the mechanics which facilitate movement in fossil species, we first must have a comprehensive understanding of the unpreserved musculature, including surface attachment sites, volume and configuration (i.e. ‘this muscle's belly overlays that muscle's belly'). Such a challenging task was recently addressed by Demuth *et al*. [18] in which three-dimensional muscles were digitally reconstructed for an extinct archosaur, and then their new method was validated with data from comparative extant archosaurs and then for mammals (specifically, primates) using polygonal modelling. In this approach, a three-dimensional model of each muscle in a body segment is created, guided by comparative data from analogous extant species. The respective entire musculature configuration within an animal's body segment is composed, while the entirety of the attachment site is digitized, not just a singular landmark. After which, a line is automatically ‘threaded' through the midline of each newly created three-dimensional muscle, representing each muscle's line of action (LoA) which is appropriately configured and spaced within the limb, improving anatomical fidelity. Importantly, knowing the space that a muscle will occupy within the body will produce LoAs that accurately represent the muscle's position, rather than LoAs which are not feasible as they might interfere with another muscle's belly. Thus, polygonal muscle reconstruction is recommended for modelling the musculature of extinct animals in which no soft tissue preserves [18]. Demuth *et al*.'s method was successful with high fidelity, shows great promise and usability, and follows a simple workflow for implementation in future studies focused on hominins—a topic of great functional debate.

The *Australopithecus afarensis* specimen AL 288-1 (commonly known as ‘Lucy'), dated to 3.2 Ma from the Hadar region of Ethiopia [23,24], is one of the most complete hominin skeletons and it has been well studied since its discovery in the 1970s [5,7,8,23,25]. AL 288-1 is predicted to have been approximately 1.05 m tall [26] with a body mass range of 13–42 kg [27–29], although the AL 288-1 specimen is typically considered to be on the lower end of the body mass spectrum of the species [30]. Today, it is generally agreed among researchers that the postcranial skeleton displays morphological features indicative of bipedality [31–35], and has thus been the focus of previous biomechanical assessments of muscle recruitment during movement [5,7,8].

The AL 288-1 specimen exhibits many anatomical features that differ to humans [23], but typically the pelvis and lower limb (henceforth referred to as just ‘limb' for brevity) anatomies have received more direct research attention, as these elements are directly correlated with this species' locomotory functionality. Such anatomical differences have polarized previous debates regarding the capability of *Au. afarensis* to walk bipedally with an erect limb [36–39]. AL 288-1 has a wider pelvis and relatively shorter legs than humans [23,25,32], which is thought to have effected muscular leverage [1,6,40].

While it is generally accepted today that AL 288-1 probably walked bipedally, questions still persist regarding the capability and frequency of such movement. Computer-based three-dimensional musculoskeletal models can be used to compute muscle function [14,16,41], such as the calculation of moment arms [9,13,15]. A muscle's moment arm is defined as the perpendicular distance between the LoA and the joint axis, representing the effectiveness with which the force produced by a muscle generates moments (or torques) at the joint(s) crossed by the muscle. A longer moment arm indicates greater muscular capabilities in which the muscle can span a greater range of its force–length curve for a given joint motion. If the moment arm is longer, then the muscle is capable of shortening and lengthening more quickly for a given joint angular velocity, thus decreasing (moment arm shortening) or increasing (moment arm lengthening) the muscle's force and torque [42]. Moment arms are thus of interest to calculate for muscles associated with locomotion in the AL 288-1 specimen which differs in both pelvic anatomy and limb proportions [1,26,33–35] that probably influence muscle path and configuration. However, moment arms, while informative, tell only a part of the story. Moment arm magnitudes do not correlate with the actual moment that a muscle can produce and, as such, magnitude alone should not be used to infer locomotor capabilities [42]. Patterns and peaks of moment arms are more informative and provide a baseline for inferring similarities in limb functionality between species.

Moment arms are intrinsically linked to bone shape, size and muscular composition [42], and thus care must be taken in defining a muscle's LoA in fossil specimens for which no musculature data exists. Changes/inaccuracies in the path can produce incorrect moment arm calculations [43], necessitating new approaches beyond the use of identifying a simple straight-line LoA between two landmarks (e.g. [1]). Rather, configuration (i.e. each muscle's volume and position within a body segment relative to other muscles) must be estimated to permit improved identification of attachment sites and LoAs.

By first reconstructing soft tissue composition in the body, more accurate estimates of each muscle's LoA can be generated, producing realistic results of muscle function which are phylogenetically informed—that is, the researcher must first consider the most analogous extant species to inform upon the condition for an extinct specimen. In this study, soft tissues of the entire pelvis and limb of AL 288-1 were reconstructed using a digital polygonal muscle modelling approach which guided musculoskeletal modelling of this specimen to demonstrate the applicability of this approach. This study aims to provide a comprehensive production of muscle space filling and path designation in AL 288-1 and to make all reconstructions available for future use so that others interested in musculoskeletal modelling of hominins may use this model as a basis.

## 2. Material and methods

### 2.1. Human data

**Figure 1.**
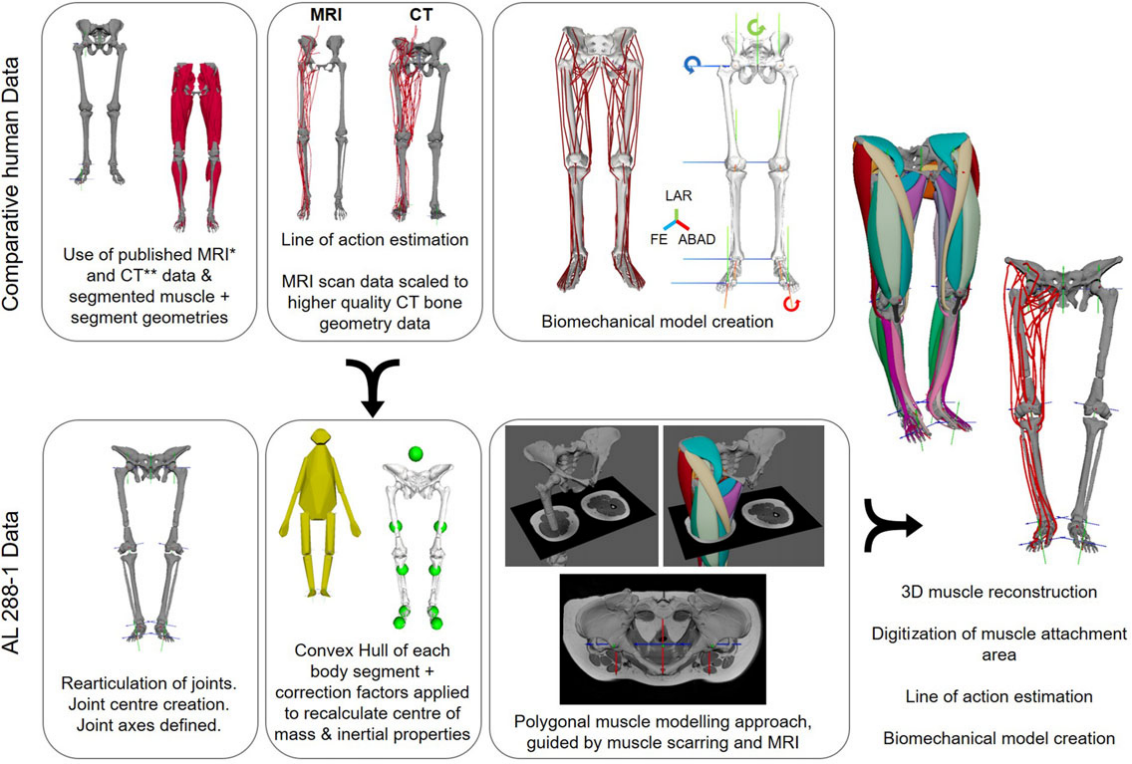
Workflow diagram outlining the process for the current study. Comparative data were collected from a male human, in which MRI data from [22] were used to guide muscle LoAs, segmental and inertial properties. The bone geometries were poor quality, necessitating these parameters to be scaled to high-quality CT data (data from [11,47]). For both the human and AL 288-1, joint centres and axes were created. A convex hull was created of each body segment of AL 288-1, of which the centre of mass and inertial properties was corrected following Coatham *et al*. [48]. A polygonal muscle approach was implemented [18], producing muscle LoAs which were implemented into the biomechanical model of AL 288-1. Note: while [22] included the *M. psoas major* (visualized here), it was excluded from the current study due to uncertainty over thorax reconstructions. LAR, long-axis rotation; FE, flexion/extension; ABAD, abduction/adduction. *[22]; **[11,48].

Through comparisons with analogous extant taxa, it is possible to infer the dimensions and extensions of the musculature of extinct species [44,45]. A modern human (henceforth, just ‘human') was selected as the closest probable analogy to the AL 288-1 specimen due to many anatomical similarities; refer to §3.4 for a discussion on the reliability of using a human versus chimpanzee base model. MRI scan data of a human's pelvis and lower limbs were sourced from an open-access repository (specimen ID: Subject03) [22]. Subject03 was an adult female, weighing 72.6 kg and 176 cm in height. All extrinsic and many—but not all (see below)—intrinsic muscles were previously segmented and available for use. Segmental mass properties were readily available for this specimen. Unfortunately, the scan quality was too poor to conduct further segmentation to capture each muscle's tendon (and thus attachment sites) and smaller intrinsic muscles, such as the hip external rotator compartment (i.e. *M. gemellus* group). For the former, each muscle's insertion and origin were estimated, guided by muscle scarring and published dissection data for the right limb [46]. Dissection data guided the latter to produce estimated muscle paths. A LoA was created for each muscle (figure 1). All right muscle LoAs were mirrored to the left side, using the central location of the pelvis (location: 0,0,0) [49,50]—described below.

The bone geometries of this specimen were low quality, and thus higher quality bone geometries of an adult male were sourced from another study to better visualize bone epiphyses for the shape fitting procedure (see below), specimen ID: TLEM2-CT [11,47]. This specimen weighed 84 kg; height was not recorded.

Using the bone geometries from TLEM2-CT, joint centres and joint coordinates systems (JCSs) were created and implemented via a shape fitting procedure [16,17,49–51]). Further details are provided in electronic supplementary material, S1. JCSs were established for the pelvis, hip, knee, ankle (the talocrural joint) and second metatarsophalangeal (MTP; other foot joints not modelled) joints [49,51,52]. The hip was permitted to move along three degrees of freedom (DOF): flexion–extension, adduction–abduction and internal–external rotation; no translational DOFs were permitted [50]. The knee, ankle and MTP joints were permitted to move along just one axis: flexion–extension/dorsiflexion and plantarflexion for the ankle. The *X*-axis was abduction/adduction, *Y*-axis was long-axis rotation, and *Z*-axis was flexion–extension, with the coordinate system shown in figure 1. Further details pertaining to set-up can be found in electronic supplementary material, S1.

The patellofemoral joint was not modelled to minimize modelling assumptions in the comparative AL 288-1 specimen, in which there is no patella recovered (note: a scaled patella is present in the AL 288-1 muscle reconstruction purely for aesthetic purposes and to assist in muscle insertion points; it is non-functional in the OpenSim model). By modelling the knee as a single joint (no patella) fixed in position, it is acknowledged that the knee results will be simplified. The implications of this are investigated in the electronic supplementary material, S1. Similarly, no internal/external rotational capability was modelled here despite rotation being key to the ‘knee locking mechanism' in human bipedality [53] due to the heavily damaged condyles in AL 288-1. Here, it was deemed unsuitable to include additional DOFs in the knee as it was not possible to model ‘smooth’ surface movement of the femoral condyles to the tibial articular facets. The open-access nature of the presented data will permit future researchers to easily ‘unlock' this DOF to extract this information if desired.

Muscle masses, LoAs and segmental properties were first calculated/created for Subject03 [22] and then applied to the high-quality TLEM2-CTmodel. The bony geometries of these specimens aligned well (i.e. the bone geometries matched in shape and length), and thus no scaling was required.

The specimen was set up in the ‘neutral posture' [50], although in other studies these poses may differ [17,49]. In this pose, all joint angles were set to 0°,0°,0° [52] from which all rotational movement deviates, with the joint axes permitting movement along each DOF. All movement deviates from this neutral posture [50], allowing the study and data to be comparable between subjects and reusable by future researchers.

Code provided by Bishop *et al*. [17] was used to create a model in OpenSim 4.3, permitting the computation of muscular moment arms over a range of joint motion [9,10,13,20,41]. Each musculotendon unit (MTU) was reconstructed with reference to the muscle LoAs, as defined above. In total, this reconstruction produced 36 MTUs in the right pelvis and limb crossing the hip, knee, ankle and MTP joints—this total does not differentiate between muscles composed of multiple heads (i.e. the *M. extensor digitorum longus*). While it is not currently possible to produce muscles composed of multiple heads in OpenSim [14,41], multiple LoAs were created representing the same respective MTU, which were then subdivided according to attachment site [15] for the following muscles: *Mm. flexor digitorum longus* and *extensor digitorum longus* (four heads in total per muscle, each inserting into a respective digit). Intrinsic muscles of the foot were not modelled; rather, only ‘foot' muscles which crossed the ankle joint were included due to the sparsity of preserved foot material in the fossil specimen (see below). One muscle was not modelled. The *M. plantaris* is a thin, vestigial muscle, with variable attachments, often mistaken as a nerve by medical students [54], and missing in approximately 20% of the population and also missing in 3/5 chimpanzees [55]. It is completely absent in gorillas and hylobatids [56]. Consequently, it was excluded.

*Wrapping surfaces* and *via points* were incorporated to ensure that muscle configurations were maintained throughout motion and did not penetrate through bone [8,16,21,57], thus improving anatomical realism of the model [12,20]. Details regarding wrapping surface creation and visualization, including sensitivity analyses, can be found in electronic supplementary material, S1.

To evaluate the accuracy of the human model [58], each muscle's moment arm was computed and compared with those primarily from the ‘*gait2392 model*' located within the OpenSim Tutorial framework [59,60]. Some muscle groups were subsequently found to be disparate in shape and peak joint orientations and, to assist in model evaluation, these specific moment arms were subsequently compared with the moment arms from Charles *et al*. [22] (*n* = 10). It was found that the moment arms produced here shared an affinity with the moment arms from Charles and colleagues, with discrepancies of the *gait2392* muscle attributed to small modelling differences, which are described in detail in electronic supplementary material, S1. Overall, the moment arms presented in the current study were considered representative of human movement, thus permitting comparative assessments between the human and AL 288-1.

### 2.2. AL 288-1 data

**Figure 2.**
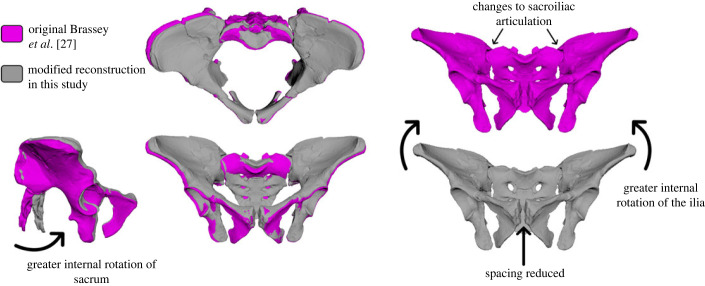
Here, *Brassey et al*. [27] reconstructed pelvis (shown in purple) was modified (shown in grey), in which the sacroiliac and pubic joints were rearticulated. Specifically, the distance between the ischiopubic ramus was reduced and both *Os coxae* were internally rotated, thus improving sacroiliac articulation.

The freely available AL 288-1 reconstruction was used [27], alongside other bony elements (humerus, scapula, proximal tibia and distal femur) that were freely sourced from http://www.elucy.org. Prior to use, modifications were first made to the reconstruction of the pelvis, in which the sacroiliac and pubic joints were modified to represent improved articulation. This was deemed a necessary step owing to potential disarticulation of pelvic joints which might not reflect biological reality [50]. Specifically, the distance between each of the ischiopubic rami were reduced and both *Os coxae* were internally rotated, thus improving sacroiliac articulation while maintaining adequate spacing for articular cartilage [27,50]. All modifications were made respective to the centre of the pelvis (previously defined by Wiseman *et al*. [50]) and thus any changes to the left side were mirrored to the right side (figure 2).

The tibial shaft was not previously reconstructed [27], but was included here as a simple, non-functional cylindrical shape bridging the gap between the proximal and distal components to facilitate muscle attachments in this region. The length of the shank segment was not modified from the original reconstruction [27], owing to previous sensitivity analyses which found that moderate changes in shank length can be ignored [8]. This recreated mid-segment has no functional significance and is included here for purely aesthetic reasons.

AL 288-1 is missing a foot. A composite *Au. afarensis* foot model was created using foot bones from AL 333 scaled to the segmental lengths of AL 288-1. All available pedal remains from AL 333 were used, while unobtained components (the phalanges) were acquired from the human. Scaling of the phalanges to AL 333 was simplistic and subjective, and involved uniformly scaling all phalanges by the same factor associated with facet articulation. It is acknowledged that this might introduce errors into muscles which attach to the distal phalanges. The insertions are ambiguous and might be either too large or small, thus influencing the muscle's distal volume, or insertions which might be too cranially or caudally located, thus influencing muscle-tendon length. In both scenarios, these errors are very likely to be only a few millimetres from reality owing to the small size of the australopith foot and assumed small size of the phalanges.

Using the same approach detailed for the human above to ensure direct comparability, a biomechanical model of AL 288-1 was created. A convex hull approach [27,48] was implemented in Matlab 2021a (Mathworks, Natick) of each body segment. The produced hulls were used as a proxy for estimating body masses, centre of masses (COM) and inertial properties in OpenSim using code provided by Bishop *et al*. [17]. A correction factor was applied to each of these estimates to correct for issues in the convex hull approach which probably under-calculates segmental mass [48]. This approach improved all segment COM and inertia estimates (see electronic supplementary material, S2), thus minimizing potential issues with subsequent moment arm predictions [8]. While COM and inertial properties do not influence moment arm calculation (see below), they were included here to standardize the manner in which musculoskeletal models of AL 288-1 (and by extent any other hominin) are constructed in the future, and also to provide the raw data for other researchers to construct a model (see below). Segmental masses were used to normalize muscle mass predictions (see below).

### 2.3. Polygonal muscle reconstructions

Obviously, it is impossible to examine muscles in a fossil via dissection or imaging techniques, and many muscle scarrings on the bones were non-identifiable. It is assumed here that the muscle attachments were most similar to those of a human rather than other analogous species owing to greater anatomical similarities [5]. However, it is acknowledged that this probably introduces some form of error, so the following is best classed as an ‘informed estimate' of muscular configuration.

A three-dimensional polygonal muscle modelling approach was implemented in Autodesk Maya 2022 (Autodesk Inc., San Rafael, CA), following the method established by Demuth *et al*. [18]. This method creates volumetric reconstructions of MTUs. Here, these reconstructions were guided by muscle scarring where visible and also by previously published cross sections of MRI scans of a human [22]. For muscles in which no scarring on the bone was visible, muscle attachment was estimated based upon published dissection data from *Homo sapiens*, and loosely by data from *Pan troglodytes* [55]. To assist in this process, muscle attachment site ‘maps’ of AL 288-1 were created and are provided in electronic supplementary material, S3a. The polygonal muscle modelling approach and implementation is described in detail in [18], but in brief:

1. Each muscle's origin and insertion were identified and digitized. The centroid of the attachment site provided a central location for the starting point of the subsequent LoA.
2. Rows of polygon edges were extruded from each of the digitized surfaces and then united to create a singular polygonal mesh, representing a muscle's shape.
3. The new polygonal muscle's extent and configuration within the body were guided via cross-sectional data of humans, scaled to AL 288-1's diaphyseal shaft diameter [18]. Owing to pelvic morphological distinctions between a human and AL 288-1 [25,33], it was not possible to use cross sections in the pelvic compartment due to misalignment; instead, these were only used for the thigh and shank compartments.
4. After all muscles in a body compartment were created (i.e. all thigh musculature), remaining ‘gaps' (i.e. spaces between the muscles) were removed by ensuring that each muscle's belly was flush against each other, replicating biological reality. Neurovascular bundles, fasciae and fasciae septa were not modelled, but small remaining gaps were left to represent such tissues where they were visible in the cross sections. Owing to morphological differences in the pelvis and limb of AL 288-1 compared with those of a human [33,34], this resulted in muscle configurations that did not precisely match the cross sections, a limitation acknowledged by Demuth *et al*. [18]. Nevertheless, the cross sections guided and informed muscular composition, i.e. constrained their reconstruction.
5. A LoA was automatically ‘threaded' through each muscle's centroid [18], and then fed into an OpenSim 4.3 biomechanical model, following the same procedure described above for the human.

While some chimpanzees have a *M. scansorius*, this is missing in humans and is instead fused with the *M. gluteus minimus* [55,56]. Here, the human form is assumed and was not included in the AL 288-1 model (see Hogervorst & Vereecke [61] for an overview of musculature differences between a human and chimpanzee).

An interactive three-dimensional scene containing all polygonal muscles, LoAs, example cross-sectional data, JCSs and bony geometries (including the modified pelvis) is freely provided (see electronic supplementary material, S3), in which the polygonal muscles (including their masses, configuration, origins and insertions) can be examined in detail and used in future research and/or outreach activities. Please note that the meshes of the proximal tibia and foot bones are not included due to copyright restrictions (see elucy.org for access to the tibia; foot bones described by DeSilva *et al*. [62]).

### 2.4. Muscle mass calculations and sensitivity analyses

Muscle masses for both species were calculated assuming a standard tissue density of 1060 kg m^−3^ [63] and were subsequently compared between species. Owing to differences in body size, both sets of muscle masses were normalized by the respective segment mass (electronic supplementary material, S4; see [13]). Notably, Subject03's muscles are partially missing their tendons due to scan data quality. As such, their total muscle-tendon masses are probably an underestimate of their true size because only belly mass is calculable. Here the lack of tendon mass contributes only to a potentially underestimated total limb mass and has no influence on the biomechanical outputs presented. To account for such underestimates on total muscle mass, sensitivity analyses were computed which increased each muscle's mass by 10% and then by 15% to establish the overall influence which tendons might have on total mass—these values are greater than the 1–2 s.d. used in sensitivity analyses on extant species by Demuth *et al*. [18] and thus ensure the full spectrum of potential muscle mass underestimation will be captured. The values here were arbitrarily selected as there is no ‘one size fits all' ratio of tendon to belly mass for muscles. This approach was repeated for the predicted segmental masses of AL 288-1 in which the convex hull approach was applied directly to bony segments, which might have underestimated true compositional mass. The segment masses were increased by 10% and then 15% to determine how underestimates may affect predictions on which species had relatively greater muscle mass per body segment.

### 2.5. Moment arm calculations

To test how muscle moment arms operated throughout each joint's range of motion (flexion–extension, abduction–adduction, long-axis rotation), MTU moment arms were calculated in OpenSim 4.3 [10,41] for AL 288-1 and the human and subsequently compared. To determine if muscle moment arms peaked at the same or different limb postures between the species across each joint's range of motion, moment arms per muscle group (e.g. hip extensors) were summed and then divided by the sum of the peak moment arm for each specific muscle, as in Hutchinson *et al*. [14] and Wiseman *et al*. [16].

The sensitivity of placing the origin and insertion of each muscle was tested by moving each attachment point up to 1 cm medio-laterally and antero-posteriorly, or proximo-distally for muscles which attached to the shafts of bones (four translations per muscle). Such method follows previous studies that tested the sensitivity of defining an attachment site in extinct species [19]. To assess the sensitivity of attachment sites, moment arms were calculated for each change in attachment location and compared with the original calculation. Full details are provided in the electronic supplementary material, S1.

The influence of the dimensions of a wrapping surface upon moment arm production was also assessed in which each wrapping surface's dimensions were changed by ±2 cm (for example, if the wrapping surface was a cylinder, then the radius was increased/decreased by ±2 cm). This value was arbitrarily selected in which changes greater than 2 cm produced wrapping surfaces that were located far from the bone's surface, resulting in unrealistic muscle paths. The moment arms for all muscles which crossed a particular wrapping object were plotted for comparison. Full details are provided in the electronic supplementary material, S1.

Finally, due to (i) a small sample size (*n* = 1 fossil specimen), (ii) identified moment arm variability within human models, (iii) slight differences due to wrapping surface dimensions, and also (iv) small differences owing to attachment site location, Monte Carlo analyses were computed of the summed moment arms to provide simulated error margins. Monte Carlo simulations were computed in Matlab 2021a across 1000 iterations using code by Wiseman *et al.* [16] in which each muscle's moment arm was randomly perturbed. For a given muscle, each moment arm curve (i.e. the magnitudes) across each joint's range of motion was perturbed by a singular, randomly assigned value that produced smooth curves, rather than perturbing each timestep independent of preceding and successive timesteps. The values for each muscle were permitted to deviate up to ±20% from their original values (assuming a random uniform distribution), based upon an average of maximally varied moment arms extracted from previous studies [16,57,64]. This method produces a ‘simulated error margin', in which our sample data are expected to deviate within the envelope to account for the aforementioned potential issues.

## 3. Results

### 3.1. Pelvis modifications

The modified pelvis reconstruction differed in the following ways: there was an internal rotation of both *Os coxae*, and both the sacroiliac and pubic joints were rearticulated to minimize joint spacing (figure 2), which was larger in the Brassey *et al*. [27] reconstruction than in other reconstructions [25,33,65]. The new bi-acetabular diameter is 118 mm, in line with previous estimates [25], but conflicting with that of Brassey *et al.* [27] who estimated 114.1 mm. Greater internal rotation of the *Os coxae* increased the subpubic angle from 77° [27] to 81°, identical to the subpubic reconstruction of Tague & Lovejoy [25]. There are other pelvis reconstructions which do differ slightly [66], although to the best of the author's knowledge, three-dimensional models are not available or open access for direct comparisons. The newly modified pelvis is freely provided (electronic supplementary material, S3).

### 3.2. Polygonal muscle reconstructions

**Figure 3.**
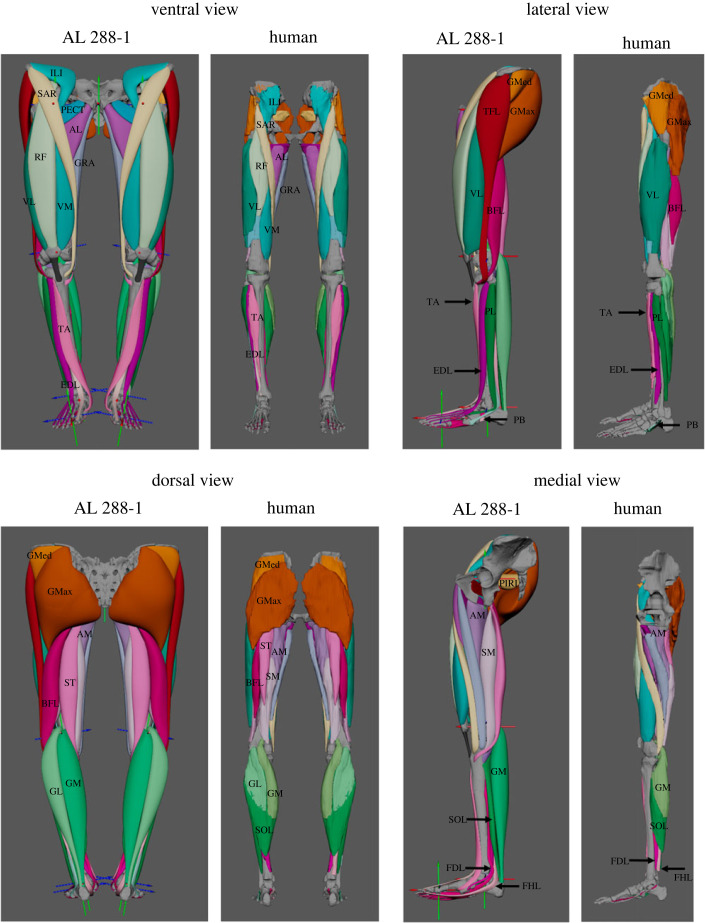
Completed views (ventral, dorsal, lateral and medial) of the polygonal muscle modelling approach in AL 288-1, in which 36 muscles were created per lower limb—this total does not differentiate between muscles composed of multiple heads (i.e. the *M. extensor digitorum longus*). The polygonal muscles of AL 288-1 are shown in comparison to three-dimensional muscles of the human which were segmented from MRI scan data. Intrinsic muscles of the foot were not modelled; rather, only ‘foot' muscles which crossed the ankle joint were included due to the sparsity of preserved foot material. See electronic supplementary material, S2 for a diagram illustrating muscle origin and insertions in which the colours correspond to the muscle colours used here. Full muscle configurations shown here, refer to electronic supplementary material, S3 to visualize deep musculature.

In total, 36 muscles were created per limb (figure 3 and table 1). Sensitivity analyses were computed on each LoA's attachment site and the influence of wrapping surface dimensions (see electronic supplementary material, S1). The results of the sensitivity analyses demonstrated that differences in both attachment site location and wrapping surface dimensions are minimal, with the few differences found to be within the variability of human musculature. Therefore, it is recommended to use simulated error margins if the model is created to represent a species or a population within a species (i.e. not subject-specific). These reconstructions are considered the ‘best informed scenario' with the available skeletal material.

**Table 1.** Muscles included in this study and their abbreviations. Muscles loosely ordered proximally-distally.

abbreviation	muscle name	abbreviation	muscle name
AB	adductor brevis	BFL	biceps femoris long head
AL	adductor longus	BFS	biceps femoris short head
AM	adductor magnus	VI	vastus intermedius
GemInf	gemellus inferior	VL	vastus lateralis
GemSup	gemellus superior	VM	vastus medialis
ObtExt	obturator externus	MG	medial gastrocnemius
QF	quadratus femoris	LG	lateral gastrocnemius
TFL	tensor faciae latae	PB	peroneus brevis
GMax	gluteus maximus	PL	peroneus longus
GRA	gracilis	SOL	soleus
GMed	gluteus medius	TA	tibialis anterior
GMin	gluteus minimus	TP	tibialis posterior
ILI	iliacus	PECT	pectineus
PIRI	piriformis	POP	popliteus
RF	rectus femoris	EHL	extensor hallucis longus
SAR	sartorius	FHL	flexor hallucis longus
SM	semimembranosus	EDL_DIGITS I-IV	extensor digitorum longus
ST	semitendinosus	FDL_DIGITS I-IV	flexor digitorum longus

The reconstructed muscles of AL 288-1 were most different to those of the human in the pelvic compartment, while other regions of the lower limbs were more similar. Increased flaring of the pelvis [25,27,33] resulted in many muscles' pelvic attachments being more transversely or frontally positioned. This had a direct impact on MTU volume and length. For example, the *M. sartorius* had a more transversely positioned origin in AL 288-1 than that of a human (the yellow polygonal muscle crossing the thigh in figure 3; see also electronic supplementary material, S3). This muscle crosses over the front of the thigh, inserting into the superior medial tibial shaft, near the tibial tubercle. A more transversely positioned origin resulted in this muscle's belly becoming elongated in AL 288-1 relative to a human, which might have affected hip flexion capacities. Unfortunately, the powerful hip flexor iliopsoas was not modelled here due to a lack of preserved thorax material in this specimen needed for the muscle's origin. Thus, conclusions regarding the effectiveness of hip flexion in AL 288-1 are partly limited because this muscle is missing from the model. The iliopsoas is the strongest hip flexor in humans [53], and it is likely that this muscle also contributed substantially to hip flexion in AL 288-1 too, assuming that primate muscles are highly evolutionarily constrained [44,45].

Other differences were found in the deep gluteal region, specifically the *Mm. gemellus inferior* and *superior* muscles (and by extent, the *Mm. quadratus femoris*, *piriformis* and *obturator externus*; see electronic supplementary material, S3). This external hip rotator muscle group originates from various aspects along the ischium (with the exception of *M. piriformis* which instead originates from the sacrum) and inserts into the medial aspect of the greater trochanter and trochanteric crest. AL 288-1 has a wider pelvis [25] than a human, resulting in the distance between these muscles' origins and insertions being relatively greater in AL 288-1 than a human. However, results of this specific muscle group should be cautiously interpreted as each muscle's attachment site was solely inferred based upon human and chimpanzee dissection data [46,55,56]. Poor quality MRI scan data of this compartment negated imaging guidance of the muscle path during the three-dimensional modelling process.

The AL 288-1 pelvis was relatively shorter than the human pelvis [1,33], resulting in many muscles' LoAs being more transversely orientated, rather than exhibiting a strong coronal orientation as in a human. For the *M. piriformis*, the LoA was almost entirely transverse in AL 288-1, rather than having an oblique angle as in a human [22]. The shortness of the pelvis further affected the LoA of the *Mm. gluteus minimus* and *medius* which reduced the relative lengths of these muscles, but the wideness of the pelvis resulted in a transverse expansion of the attachment site.

Owing to the configuration of the entire gluteal region (superficial and deep), it was impossible to model the *M. gluteus maximus* as having a component of its fibres attaching to the ischium, as previously suggested by Berge [1], although this is in line with other studies [32,33]. This muscle must have had a similar origin in the pelvis to that of a human because the path of the *M. piriformis* would have prevented the *M. gluteus maximus* from occupying a space flush to the ischium, while other muscles' volumes further prevented this muscle from extending deeper into the compartment. However, gluteal muscle scarring was poor, and the polygonal approach was an estimate, so this should be duly noted as a potential limitation.

AL 288-1's femur was short relative to the pelvis height in comparison with a human [1,4,32–35]. Evidently, length of the quadriceps femoris muscle group was relatively shorter in AL 288-1 than the human (knee extensors; *Mm. vastus medialis*, *vastus lateralis*, *vastus intermedius, rectus femoris*). The relatively longer human femur resulted in longer muscle lengths.

Polygonal reconstructions of the adductor group (*Mm. adductores magnus*, *longus*, *brevis*) demonstrated that these were more ventrally positioned than in humans. The attachment site of the *M. adductor magnus* was similar to that of a human owing to distinct faceting and a transverse ridge [33] on which this muscle originated, which is a feature that is missing in non-human apes [39].

The AL 288-1 condyles were damaged and poorly preserved. Therefore, modelling errors may exist in muscles which wrapped around this feature (e.g. the *M. biceps femoris*). Specifically, the *Mm. popliteus*, *gastrocnemius lateralis* and *medialis* all originated from this region and might have the greatest source of error in the identification of attachment sites (figure 3; see also electronic supplementary material, S3). AL 288-1 had a shorter shank than a human [8], and as such the main differences in musculature in the lower leg were due to length rather than spatial configuration.

The AL 333 foot [62] was used as a composite to ‘complete' the lower limb. Differences in musculature to a human (inferred from moment arms; see below) probably related to curvature of the metatarsals [39], morphological differences in the convexity of the cuneiform hallucal facet which probably influenced metatarsal orientation, and a less pronounced longitudinal arch [62,67].

### 3.3. Muscle mass comparisons

**Figure 4.**
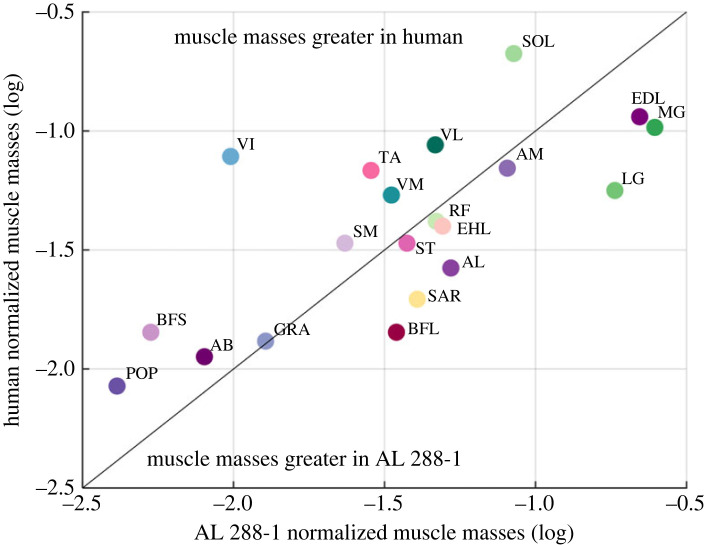
Logged normalized muscle mass calculations of AL 288-1 plotted against logged normalized muscle masses of the human. Each muscle mass was normalized by the respective segment mass in which the muscle's belly resides. Coloured data points correspond to the colours of muscles in figure 3, the three-dimensional polygonal muscles available for download in electronic supplementary material, S3, and also the muscle map diagrams in electronic supplementary material, S3a. All other muscle mass calculations for AL 288-1 can be found in electronic supplementary material, S4.

Muscle masses were compared between AL 288-1 and the human (figure 4; electronic supplementary material, S4). Sensitivity analyses increased muscle masses in the human by 10% and by 15% to account for incomplete total muscle mass (see Methods), but these analyses did not influence the overall trend in the results (electronic supplementary material, S4). Ten muscles in AL 288-1 were between 10% (*M. semitendinosus*) to 106% (*M. gastrocnemius lateralis*) greater than in the human (figure 4), the latter of which had the greatest discrepancy by a factor of 3.3. Muscles which were smaller in AL 288-1 were mostly in the thigh compartment, in which the differences between species ranged between 2% (*M. gracilis*) and 156% (*M. vastus intermedius*).

For the 20 muscles for which these data were available, muscles constituted 73.76% of the total mass of the thigh in AL 288-1, but just 49.76% in the human. In the shank, these muscles accounted for 51.02% of the total segment mass in AL 288-1 and 49.14% in the human; and in the foot, the muscle masses accounted for 52.58% of the total segment mass in AL 288-1 but just 15.46% in the human (unsurprising due to a lack of pedal musculature present). The lack of tendon and deep musculature in the scan data of the human probably accounts for some of the remaining mass, while bone mass and also fatty deposits might compose the rest of the segmental mass [68]. If all thigh musculature is included for AL 288-1, then the muscles account for 78.99%, indicating that the AL 288-1 thigh was probably composed of more muscular tissue than fatty tissues.

Increases in segmental and/or muscle mass (i.e. the sensitivity analyses) found that 75% of muscles were smaller in AL 288-1. If muscle masses were compared between the non-corrected segmental masses [48], then muscle masses would have been greater in AL 288-1 (16 muscles, ranging from 22% to 167% of relative muscle), in which some muscles would have been greater by a factor of 11.7 (*M. biceps femoris longus*), indicating that correction factors are indeed required for realism and uncorrected convex hull models are not sufficient and considered implausible.

### 3.4. Moment arms

**Figure 5.**
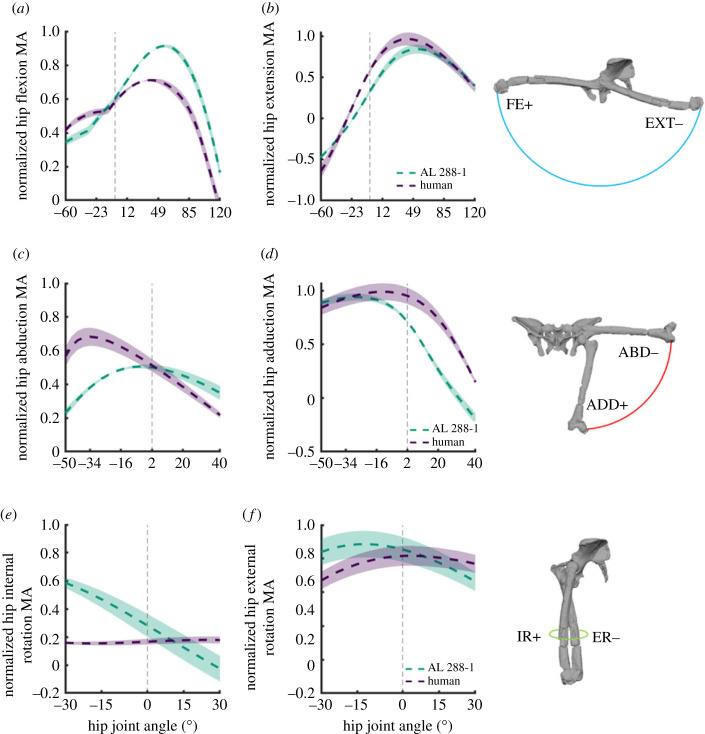
Sum of flexor (*a*), extensor (*b*), abductor (*c*), adductor (*d*), and rotator (*e*,*f*) moment arms normalized by the sum of the maximal moment arm for the associated motion, plotted against hip flexion–extension (*a*,*b*), abduction–adduction (*c*,*d*) and internal and external rotation (*e*,*f*). The vertical grey dashed line denotes joint position 0. Example joint rotations provided.

The hip flexor group peaked at approximately 55° flexion in AL 288-1, whereas the human group peaked instead at approximately 30° flexion with a lower magnitude (although, it should be reiterated that inferences based upon magnitude are somewhat redundant without associated moment-generated capacity, which are not reported here) (figure 5*a*). More flexed postures (positive rotations; i.e. towards 120° flexion) have a reduced moment arm capacity in both species, whereas more extended postures (negative rotation; i.e. towards −60° extension) also have a reduced moment arm capacity, but with a greater magnitude in both species than that of flexed postures, although such poses are not observed during normal gait [69].

The summed extensor moment arms were similar between species, whereby the moment arms increased as hip extension was decreased (figure 5*b*). Both peaks were during hyper-extended poses of −60°, beyond normal walking gait range [69]. While these poses are not observed during habitual walking activities, they are still osteologically feasible. For both species, the moment arms steadily decreased as the hip moved towards a flexed limb, with an increase during hyper-flexed postures.

The hip abductors were different between species (figure 5*c*). In the human, the moment arms peaked at approximately 43° abduction at a greater magnitude than AL 288-1 before decreasing as the hip moved into adducted poses (positive rotations). In AL 288-1, such changes were less pronounced with a peak at approximately −10° abduction after which the moment arm exhibited a more gradual reduction as the limb became more adducted. The hip adductors were also similarly patterned, with a gradual increase as abduction decreased and a decline during adducted poses (figure 5*d*). The peak in AL 288-1 was at approximately −30° and in the human it was approximately −10°.

The hip internal rotator group differed in moment arm patterns and peaks (figure 5*e*). The human moment arms had a near-plateau for most joint angles across its range of motion (ROM), with a slight increase during internally rotated postures at approximately 15°–30° (positive rotations). Contrastingly, the summed moment arm in AL 288-1 steadily decreased throughout rotation, with a peak during hyper-externally rotated postures instead (negative rotations). The summed moment arms of muscles permitting external rotation were somewhat comparable between species in terms of magnitude (figure 5*f*). The human exhibits an increased capacity of the moment arm as the hip is moved into internally rotated postures, while the AL 288-1 moment arm reduced in capacity during internal rotation. The external rotators for both groups were greater in terms of magnitude than that of the internal rotators. However, the error margins for both groups considerably overlapped, and thus it is not advisable to postulate on functional differences.

**Figure 6.**
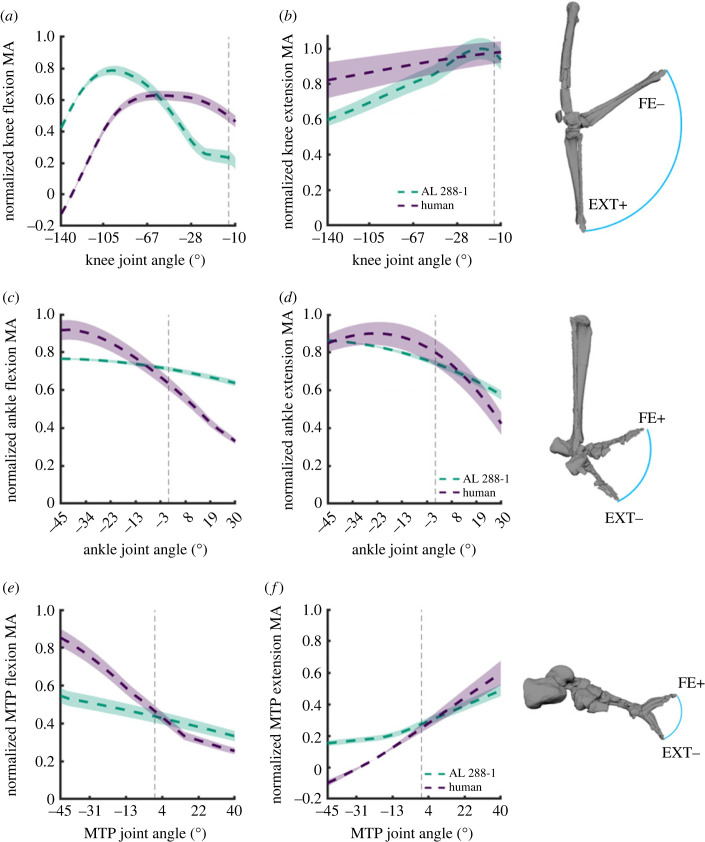
Sum of flexor (*a*,*c*,*e*) and extensor (*b*,*d*,*f*) moment arms normalized by the sum of the maximal flexor and extensor moment arms, plotted against knee flexion–extension (*a*,*b*), ankle flexion–extension (*c*,*d*) and MTP dorsiflexion (flexion; positive rotation)–plantar flexion (extension; negative rotation) (*e*,*f*). The vertical grey dashed line denotes joint position 0. Example joint rotations provided.

There were differences in the summed moment arms of the knee flexors (figure 6*a*). The magnitude was greater in AL 288-1 during the highest amounts of knee flexion (negative rotations) and peaked at more flexed positions (approx. −95° flexion), before decreasing in value in more extended joint positions after which the moment arm plateaued at approximately 21° flexion until full knee extension (0°; positive rotations). In the human, the moment arm steadily increased during hyper-flexed postures before peaking at approximately −80° flexion which plateaued for much of knee flexion until approximately 19° flexion. This provided sustained leverage during these flexed knee poses, after which there was a short reduction in moment arm length during knee (hyper-)extension (0°+).

The summed knee extensor moment arms were somewhat similar between species, although there are notable differences in terms of magnitude during hyper-flexed knee postures. The error margins for both groups considerably overlapped towards extended postures, and thus the peak moment arms can be considered comparable (figure 6*b*).

Both the summed moment arms for the ankle flexors and extensors had somewhat similar patterns, but the moment arm magnitudes differed for both joint rotations during more plantarflexed postures. In AL 288-1, both the dorsiflexor and plantarflexor moment arms were almost at a plateau throughout the joint's range, with a reduction in moment arm length towards dorsiflexed postures (negative rotations) for both the dorsiflexors and plantarflexors (figure 6*c,d*). In humans, the dorsiflexion curve had a steep decline in magnitude during dorsiflexed postures, while the plantarflexion moment arms steadily increased as the joint moved from a dorsiflexed position into plantarflexed rotations (positive rotations). Comparable patterns were observed for the MTP flexors, although the human once again exhibited a steeper decline in magnitude during plantarflexed postures (figure 6*e*). The summed moment arms for the MTP extensors increased during extended postures (negative rotations) and peaked during flexed postures (positive rotations) (figure 6*f*). The error margins overlapped during dorsiflexed postures, with differences in magnitude during plantarflexed postures.

## 4. Discussion

This study presents a polygonal reconstruction of musculature in the AL 288-1 pelvis and lower limb. Comparisons of muscle masses between the human and AL 288-1 indicated that while muscle masses were smaller in AL 288-1's thigh than in the human, overall the muscles of AL 288-1 constituted a greater composition of muscle mass within the body segment in which the human thigh was probably less muscular and more fatty. This is similar to what we see in bonobos, an active, arboreal species [68]. AL 288-1 possibly exploited a range of habitats and locomotory repertoires and, thus, retained greater relative muscle mass than a human in some muscle groups (i.e. the hip adductors), possibly corresponding to muscle power capabilities associated with different types of movement.

Muscle moment arms were investigated and compared between the australopith and human. Moment arms are inherently driven by anatomical differences. For example, AL 288-1 has greater flaring in the ilia and a relatively longer femoral neck. A longer femoral neck increases the distance between the insertion point of the muscles which insert into the greater trochanter (e.g. the adductor muscle group) to that of the hip joint centre (i.e. the point of rotation). A longer moment arm is indicative of a muscle generating greater torque [42], but it should be stressed that the respective muscle origin point also influences moment arm length. Evidently, a trade-off exists in the AL 288-1 specimen between pelvic anatomy and femoral neck length in which skeletal configuration has resulted in both species having mostly comparable summed moment arms across the hip muscle groups. Therefore, it is possible to postulate that a longer femoral neck in AL 288-1 assisted in producing effective leverage in the pelvic/hip muscle region necessary for range of movement, including upright walking.

Anatomical differences in the pelvis and shorter segment lengths will influence muscular leverage [42,43] by impacting their length and path, but these differences do not appear to be impeding the ability to flex, extend, adduct and externally rotate the hip with respect to the human. Differences in moment arm magnitudes and peaks associated with a range of different joint orientations could represent the repertoire of motions that AL 288-1 probably employed, perhaps ranging from bipedal walking to climbing [3,38,70,71], but broad similarity in moment arm patterns precludes any further statement. Declarations of functional differences will only be possible with the inclusion of moment-generating capacity, which should be targeted in future studies.

The hip extensors of AL 288-1 were similar to the human, which implies functional affinities. A shorter femur relative to pelvis height alongside a posterior expansion of the AL 288-1 ilium [25,33] enabled the gluteal compartment (specifically, the *M. gluteus maximus*) to extend the hip in a comparative way to a human, thus permitting human-like bipedal leverage. This is dissimilar to the crouched bipedal posture of a chimpanzee in which this muscle is not well-suited for hip extension owing to a more coronal orientation of the ilium instead [6,20,40,61,72]. This finding is in contrast to earlier studies which used simple linear action lines to estimate function [1], but in line with other studies that used advanced computational models [5,8]. Evidently, developments in computational methods have improved estimates of muscular leverage in extinct species, and similarity between the model presented here with previous biomechanical models (e.g. [8]) stresses the usefulness of polygonal modelling in advancing our knowledge of muscular configuration within the limb and of leverage. Importantly, polygonal muscle modelling clearly shows where space must be occupied by a muscle, and where LoAs would not be feasible due to interference with another muscle. For example, if the *M. gluteus maximus* has indeed occupied a space flush to the ischium in AL 288-1 as Berge [1] argued, then this would have ignored the space occupied by other muscles and any inferences regarding leverage would be wrong. In reality, other muscles' bellies (i.e. the internal hip rotator group) restricted gluteal expansion, and thus the reconstruction presented here is probably on track for realism.

The ischial tuberosity—where the hamstring muscle group originates (*Mm. biceps femoris longus*, *semitendinosus*, *semimembranosus*)—has a dorsal projection in AL 288-1 relative to a human [39,73]. Pontzer *et al*. [6] argued that this reduces the lever arm capability of this group, favouring a habitually crouched posture rather than an extended limb in AL 288-1, although Kozma *et al*. [3] highlighted that the leverage of this group was still within the lower bounds of humans, which was argued to permit a human-like capability for powered hip hyperextension and walking economy. In contrast to these more recent studies, Berge [1] argued that the hamstrings must have had greater leverage in AL 288-1 than a human due to having a longer lever. Here, both the human and AL 288-1 extensor groups had a somewhat similar pattern, but different joint angles that correspond to peak moment arms and magnitudes which are in line with the findings of Kozma *et al*. [3].

The human knee is suited for midstance postures during a period when just one limb is on the ground [50]. Although the moment arms in AL 288-1 were greater during hyper-flexed postures than those observed during habitual walking activities as observed in the human, the moment arms during these walking poses (i.e. [69]) were not ineffective. This implies that the AL 288-1 knee was potentially suited to a range of movement types, beyond what we observe in humans.

During upright walking, the knee must also extend. The AL 288-1 knee extensors demonstrate the capability of producing knee extension, a necessary tool for bipedal walking. Based upon the moment arm results, AL 288-1 could have—and probably—walked and stood upright with an erect posture of the knee (i.e. full extension), supporting previous biomechanical analyses [3,5,7,8]. However, it remains unknown if upright walking was a highly frequent activity and further investigations using dynamic simulations could address this more fully.

Further down in the limb, the results do not support previous studies which argued that a shorter shank relative to humans would reduce the moment arm capability of the posterior shank compartment (*M. soleus*, *Mm. gastrocnemii lateralis* and *medialis*) [8]. Rather, there was broad similarity in the plantar and dorsiflexors of the ankle, which precludes any further statement as to differences in probable function. These results should be evaluated alongside the effectiveness of foot pronation and supination in the future, which has been avoided here due to the composite AL 333 foot used and the potential for scaling issues which might influence results.

There were some differences in the dorsiflexor and plantarflexor compartments of the foot, which could be functionally related, but potential modelling uncertainties cannot be dismissed. The degree of hallucal abduction may have been insufficiently modelled here, which might have influenced the results of muscles which crossed the MTP joint. Here, only the second digital ray was modelled which represented all digital movement, and as such there are limited inferences that can be made regarding the moment arms of muscles which cross the other digits. On the other hand, some differences in the moment arms of these muscle groups might be expected due to some possible retention of opposability [62,67,74] which would have certainly affected leverage. There was strong similarity between the human and AL 288-1 in the moment arms of the plantarflexor groups during plantarflexion. Future studies should incorporate forward dynamic simulations to test function with respect to applied forces to ascertain if AL 288-1 could have used the distal foot in a similar manner as a human and thus might have had human-like foot motion [5,9].

This study used a single LoA to represent each muscle as a simple demonstration of how the polygonal muscle reconstructions can be used to create musculoskeletal models. Greater fidelity in moment arm calculation will probably be achieved by creating numerous LoAs representing a singular muscle, stemming from the attachment sites—this is particularly pertinent for fan-shaped or broad muscles, such as the gluteal muscles. The *M. gluteus medius* is fusiform but has a broad proximal attachment which can be best represented with multiple LoAs. Such approaches have been adopted in human biomechanical studies [12,47] and might improve upon estimates of compartmental moment arms in the future. The space-filling approach used here is, arguably, the best method for producing these muscle subdivisions needed for this approach, especially for extinct taxa. Here, the polygonal muscle reconstructions provide a baseline approach for assessing muscle functionality in which all data are provided for subsequent biomechanical assessments.

### 4.1. Did AL 288-1 walk bipedally with an erect limb?

While there were many key differences in muscle capability in the pelvis and limb of AL 288-1, the moment arms were overwhelmingly comparable along many joint axes, indicating that AL 288-1 was capable of an erect stance posture, as previously suggested [5–8,50,73,75], although the efficiency and stability of such a posture should be investigated further. Some differences in moment arm patterns and joint angles corresponding to peak moment arms were expected owing to a suite of anatomical variations [26], but the overwhelming similarity in the moment arms could be argued to support previous theories of an adducted, erect limb [5,8], casting doubt upon earlier assumptions that AL 288-1 must have walked with a crouched limb posture [38,39]. However, such a statement is limited here as only moment arms were included. These results must be evaluated [58] by future estimations of moment-generating capacity because the results presented here only tell a part of the story and cannot wholly rule out the possibility of a crouched-like posture.

It is pertinent to acknowledge that the results and inferences presented here will be bolstered by future studies incorporating muscle parametrical data (fibre length, tendon slack length, pennation angle, etc.). Future studies should also appreciate that slight differences in muscle volume can influence the amount of estimated force that a muscle can generate which should be appropriately investigated in light of the muscle mass sensitivity analyses presented [76].

Finally, the body mass of AL 288-1 is considered to be on the lower end of the range of adult body sizes for australopiths [30]. There might be implications for moment arm assessments for a smaller specimen and then subsequently extrapolating such results to the species level, but this is impossible to ascertain without a larger australopith sample size. Nevertheless, the results here do demonstrate that AL 288-1 had similar moment arms upon which we can infer relative muscular function as a human (i.e. the ability to stand upright and move with an erect-style posture), so it would seem very unlikely that larger body-sized individuals would not fall into the same category.

### 4.2. How accurate is the model?

There are known problems and limitations with the AL 288-1 reconstruction, acknowledged by Brassey *et al*. [27]. Many components of the pelvis and limb in AL 288-1 are estimated reconstructions and the foot belongs to another specimen. Not all muscles retained evidence of muscle scarring, and thus some muscle origin and insertion sites had to be estimated (see Material and methods).

Muscle masses between AL 288-1 and the human were variable, with 50% of the muscles greater in the human and 50% greater in AL 288-1. Muscle mass in *Pan paniscus* (bonobos) exceeds those of humans in which relative muscle mass has generally decreased alongside increases in body fat composition in humans [68], which is the trend we see in the AL 288-1 thigh mass in which muscle mass occupied more of the body segment (78.99%) than in the human (49.76%). Perhaps it is unsurprising to find that 50% of muscle masses (or up to 75% if considering the sensitivity analyses) were greater in AL 288-1, a species which possibly exploited a range of habitats and locomotory repertoires and, thus, retained greater relative muscle mass than a human in some muscle groups (i.e. the hip adductors), possibly corresponding to muscle power capabilities.

Cumulatively, these factors lead to the acknowledgement that there are uncertainties and/or potential inaccuracies in this muscle modelling approach that cannot be circumvented without a full, well-preserved skeleton. Nevertheless, the reconstructed skeleton and the polygonal muscle reconstruction method provides good estimates of body composition, which can subsequently be used to infer locomotory capabilities.

Finally, it must be acknowledged that the human form was selected here as the basis for reconstructing AL 288-1's muscles. If another primate (i.e. a chimpanzee) was selected as the basis for reconstruction, the results would possibly have differed, although probably not by much because vertebrate musculature is assumed to be highly evolutionarily constrained [18,77–79], although see [80]. While there are some minor differences in origin and insertion locations between primate species, these differences are also observed intra-variably [55,56]. Major differences exist in the loss of some muscles in humans which are present in other great apes (e.g. the *M. scansorius*), differences in muscle mass composition, and in the parameters of each muscle (i.e. fibre length, physiological cross-sectional area). The latter is not of concern for the current study. Mass differences are directly tested and accounted for here using sensitivity analyses.

Additionally, there are some skeletal differences between a human and AL 288-1, notably in the pelvis compartment, femoral neck length, bi-acetabular diameter and limb proportions. Some differences observed in the pelvic musculature might be due to the MRI cross-section misaligning in this region, but these differences are thought to be minor because the reconstructions were guided by dissection data and vertebrate musculature is highly evolutionarily constrained [45]. More so, the AL 288-1 specimen was female and the human specimen used here was male, but with female musculature that was scaled to the male skeleton. Human pelvises are sexually dimorphic [81] but due to limitations in data availability, sexual dimorphism was ignored. This highlights a level of uncertainty and potential subjectivity in the reconstruction process. However, the numerous anatomical similarities between this species with a human and the many anatomical dissimilarities with a chimpanzee provide credence to the selection of the human as the baseline.

## 5. Conclusion

Here, a reconstruction of muscular configuration in the AL 288-1 pelvis and lower limb is presented, comprising 36 muscles and their spacing within each body segment. On the basis of the muscle leverage results, *Au. afarensis* was capable of producing an erect posture but was also capable of using the limb in a repertoire of motions beyond habitual terrestrial bipedalism, in a similar manner to chimpanzees and bonobos. As it stands, these results cannot confirm if AL 288-1 was an efficient biped, but rather that upright walking was a possibility. Moving forward, the polygonal muscle modelling approach has demonstrated promise for reconstructing the soft tissues of hominins and providing information on muscle configuration and shape filling. Future studies investigating muscular function of hominins should consider (i) the space filling within a desired body segment, such as muscle paths and masses, (ii) the importance of digitizing the entire attachment site surface to elucidate a realistic centroid, and (iii) the need to consider all muscles acting upon a body segment, not just a single muscle. An interactive three-dimensional Autodesk Maya scene of all musculature is provided alongside this paper to aid future research and perhaps even assist in human evolutionary anatomy teaching.

## Data Availability

An interactive Autodesk Maya scene is provided open access in electronic supplementary material, S3 [82]. This scene contains all polygonal muscles, the modified pelvis, MRI cross sections and LoAs, and the user will be able to fully explore all aspects of the reconstruction and use the models in any format for their own research (within CCBY 4.0 restrictions). Autodesk provides free academic/research licences to all researchers/students affiliated with a university/museum/research institute. Note that an .FBX file format is also provided which can be readily opened in other three-dimensional modelling software, retaining colours, groupings and layers.
